# Assessment of IL-6 and IL-8 Levels and Other Bio Markers in Predicting Dengue Severity Across Serotypes

**DOI:** 10.3390/pathogens15040434

**Published:** 2026-04-17

**Authors:** Kumar Sivasubramanian, Rudrappan Raj Bharath, Leela Kakithakara Vajravelu, Madan Kumar D, Jayakrishna Pamarthi

**Affiliations:** 1 Department of Microbiology, SRM Medical College Hospital and Research Centre, SRM Institute of Science and Technology, Kattankulathur, Chengalpattu 603203, India; ks1063@srmist.edu.in (K.S.); leelav@srmist.edu.in (L.K.V.); md5398@srmist.edu.in (M.K.D.); jp9860@srmist.edu.in (J.P.); 2 Department of Transfusion Medicine & Blood Centre, SRM Medical College Hospital and Research Center, SRM Institute of Science and Technology, Kattankulathur, Chengalpattu 603203, India

**Keywords:** platelet transfusion, severe dengue, cytokines, biomarkers, dengue serotypes

## Abstract

Background: Dengue fever is one of the most common mosquito-borne viral infections, with severe cases characterized by plasma leakage, hemorrhage, and multi-organ involvement. Identification of dengue serotypes and reliable biomarkers is essential for predicting disease progression and guiding timely interventions. Methods: This prospective cohort study was conducted at a super-speciality tertiary care hospital in southern India from July 2024 to July 2025. A total of 69 patients presenting with dengue warning signs were included in the study. Patients were categorized into the severe dengue group (*n* = 25) and non severe dengue group (*n* = 44). Clinical data, laboratory findings, dengue serotype, and serial serum samples collected on Days 1, 4, and 8 were analyzed to evaluate the predictive and monitoring efficacy of Interleukin-6 (IL-6) and Interleukin-8 (IL-8), and followed up till discharge. Results: Out of 69 dengue patients with warning signs, 32 dengue-positive patients were serotyped, which included DEN V-1 (31.3%), DEN V-2 (31.3%), DEN V-3 (15.6%), DEN V-4 (18.8%), and mixed DEN V-(2 + 3) (3.1%). Severe dengue patients exhibited a higher frequency of secondary dengue infection (IgG) than primary dengue infection (88% vs. 12%), with statistically significantly higher packed cell volume, hemoglobin levels, high AST levels, and prolonged activated partial thromboplastin time, as well as lower platelet counts and albumin levels. Platelet transfusion was given to 35 dengue patients, which had also resulted in significant length of stay in hospital in comparison to non-transfused patients. IL-6 and IL-8 levels were significantly elevated in severe dengue patients when compared to non-severe dengue patients on Day 1 and Day 4, followed by a decline on Day 8, corresponding with clinical recovery. However, the elevated IL-8 levels were observed to be significantly associated with longer hospital stays, indicating its potential role as an early predictor of disease progression. Conclusions: The observed co-circulation of multiple serotypes reflects the hyper-endemic pattern reported across India. Early measurement of these cytokines IL-6 and IL-8 helps distinguish severe from non-severe dengue among patients presenting with warning signs. IL-6 and IL-8 may have potential as biomarkers for disease severity. However their role in guiding platelet transfusion requires further investigation in non-severe cases and prioritizing timely management for those at higher risk of severe disease.

## 1. Introduction

Dengue is a significant public health challenge in India, marked by frequent cases and multiple outbreaks of four different serotypes (DENV-1 to DENV-4). The hyper-endemic characteristic of dengue elevates the probability of secondary infections, potentially affecting disease severity and laboratory findings [1,2]. Recent investigations from dengue endemic regions have revealed differing serotype predominance. A cross-sectional investigation documented the concurrent circulation of several serotypes [3]. Hospital-based studies conducted in Northern India (2021–2022) and other locations of North India recorded a changing prevalence of DENV-1, DENV-2, and DENV-3 [4,5,6,7]. The rise in DENV-4 during the South Indian outbreak was linked to modified cytokine expression, indicating serotype-specific immune responses [8]. Evidence from Sri Lanka further supports the correlation between the infecting serotype and changes in clinical and laboratory characteristics [9]. Numerous observational studies have demonstrated associations between dengue serotypes and severity markers, including thrombocytopenia, increased liver enzymes, and coagulation abnormalities [6,7,8,9,10]. However, individuals exhibiting warning signs still lack a thorough assessment of serotype distribution in conjunction with hematological, biochemical, inflammatory, and coagulation parameters during follow-up samples.

Cytokines play a key role in the development and progression of dengue. Interleukin-6 (IL-6) is a multifunctional pro-inflammatory cytokine that plays a role in acute-phase response and heightened vascular permeability, both of which are associated with severe dengue. Interleukin-8 (IL-8), a chemokine produced by monocytes, endothelial cells, and epithelial cells, serves as a powerful neutrophil chemoattractant and is linked to endothelial damage, thrombocytopenia, and plasma leakage [11,12]. Several research studies conducted indicate that IL6 and IL8 concentrations are markedly increased in individuals with severe dengue relative to those with non-severe cases [13,14]. An international cohort study published in 2024 demonstrated earlier elevations of IL6 in secondary dengue infections, correlating with clinical markers such as thrombocytopenia and elevated AST, highlighting the need to account for infection when interpreting cytokine kinetics [15]. Nevertheless, IL-8 thresholds have not been determined for the Indian demographic. Furthermore, the majority of existing studies are cross-sectional, concentrating on a singular time-point assessment, and fail to evaluate cytokine variations in the follow-up samples until patient discharge, hence constraining their utility for tracking therapeutic response. This study seeks to evaluate the incidence of circulating dengue serotypes and investigate their correlation with significant laboratory markers in dengue patients exhibiting warning signs, and to measure IL-6 and IL-8 concentrations in dengue patients prior to treatment and following clinical stabilization while evaluating their correlation with disease severity and therapeutic responses.

## 2. Materials and Methods

### 2.1. Samples and Study Design

**Figure 1:**
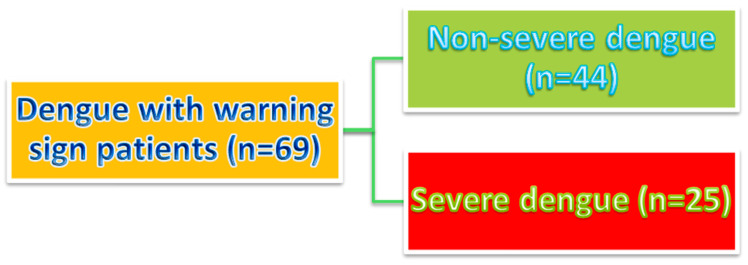
Flowchart of dengue with warning signs from the dengue hospitalization patients. **Inclusion criteria**: dengue-positive patients (>18 years age) with warning signs (positive of either NS1 or IgM) who were admitted in the super-speciality tertiary care hospital. **Exclusion criteria**: dengue-positive patients without warning signs are excluded.

This prospective cohort study was conducted from July 2024 to July 2025 at a super-speciality tertiary care hospital in southern India. A total of 197 dengue-positive adults were admitted during the study period. Of these, only 69 patients who fulfilled the inclusion criteria, namely the presence of dengue warning signs as defined by the WHO 2009 classification and the requirement for hospitalization, were selected for our study. From this, they were categorized into Severe dengue (*n* = 25) and Non-Severe dengue (*n* = 44) groups [1] (Figure 1). Dengue warning signs included abdominal pain, persistant vomiting, mucosal bleeding, fluid accumulation, hepatomegaly, and rising hematocrict with rapid decrease in platelet count, as of the 2009 WHO guidelines (1).

This data contains patient information, radiological findings, and other clinical data from the dengue patients. From the patients with follow-up samples (Day 1, Day 4, and Day 8), the hematological parameters (WBC, PCV, platelet count, and hemoglobin), liver function tests (AST, ALT, total protein, albumin, globulin, and A/G ratio), coagulation parameters (APTT, PT, and INR), inflammatory markers (IL-6 and IL-8) and dengue genotypes (dengue virus serotypes—1, 2, 3, 4) were collected and tested to determine if they had dengue with warning signs [16]. The Institutional Ethics Committee (clearance number: ECR/8812/INST/TN/2013/RR-19) approved this study, verifying that ethical norms were followed.

### 2.2. Differentiation Criteria for Primary and Secondary Dengue Infection

Primary DENV infection was defined as either NS1 Ag+ IgM- IgG- or IgM+ IgG- and secondary DENV infection as either IgM- IgG+ or IgM+ IgG+. In the presence of both IgM and IgG antibodies, differentiation is achieved using a IgM/IgG ratio cut-off value of 1.58 (Table 1) [17]. The rapid elevation of IgG levels in the initial days of sickness during secondary infection is predictive of dengue when the ratios of IgM to IgG are assessed [18].

### 2.3. Severe Dengue Classification

According to the WHO 2009 dengue classification, severe dengue is defined by two criteria: an elevated hematocrit level and bleeding and plasma leakage indicated by the presence of pleural effusion, ascites, and low albumin levels [1,19] (Table S1).

### 2.4. IL8 and IL6 Determination in Serum Samples

The IL-8 standard curve range (0–500 pg/mL) and IL-6 standard curve range (0–400 pg/mL) concentration detection by the ELISA (Enzyme Linked Immunosorbant Assay) for IL-6 (Krishgen Biosystems; Catalog No: KB1068) and IL-8 (Elabscience; Catalog No: E-UNEL-H0099) were carried out using recommended methods in the supplier’s protocol.

### 2.5. RNA Extraction and Dengue Serotypes Testing

RNA extraction was performed utilizing the HiElute Miniprep Spin Column kit (Cat. No. MB615) in accordance with the manufacturer’s guidelines.

Dengue virus genotyping was carried out with the HELINI Dengue Virus Genotyping Real-Time PCR Kit (Cat. No. 8014) following the manufacturer’s guidelines. Serum samples undergo viral RNA extraction with a validated extraction kit, with RNA eluted in 60 µL of RNase-free water. The reaction setup and amplification were conducted under prescribed settings to assure precise genotyping outcomes.

### 2.6. Statistical Analysis

Continuous variables are presented as mean and SD, and categorical data are expressed as frequency with percentage. The chi-square test was used to test the differences between categorical variables and Levene’s independent *t*-test was used to assess homogeneity of variance in continuous variables. A one-way Anova test was also performed to compare the mean values of laboratory parameters and clinical parameters from more than two categorical variables of the dengue serotypes. A *p*-value of <0.05 and <0.01 was considered statistically significant. ROC curve analysis and the Youden index (J = sensitivity + 1 − specificity) were also done to discriminate between the Severe group and Non-Severe group using the IL-6 and IL-8 levels [20]. Finally, a crosstab was performed for the risk of severe dengue. All analyses were performed using the statistical software SPSS, Version 25.0.

## 3. Results

Descriptive analysis revealed significant differences between the two groups (Severe vs. Non-Severe dengue) with respect to clinical and laboratory parameters. Key clinical and radiological findings included bleeding manifestations (64%), gallbladder wall thickening (48%) and pleural effusions (28%). Laboratory parameters such as hemoglobin (Hb) (14.3 ± 1.9 g/dL), packed cell volume (PCV) (43.3 ± 5.02%), aspartate aminotransferase (AST) (217.1 ± 243.4 U/L), activated partial thromboplastin time (aPTT) (42.4 ± 12.4 s), inflammatory markers IL-6 (297 ± 116 pg/mL), IL-8 (351.7 ± 69.1 pg/mL), and length of hospital stay (6.2 ± 1.1) were markedly elevated, and while platelet count (40,768 ± 39,267.6 cells/mm^3^) and albumin (3.4 ± 0.5 g/dL) declined in the Severe dengue group (Table 1).

**Table 1:** Descriptive data of Severe and Non-Severe group.

Variable	Severe (*n* =25)	Non-Severe (*n* =44)	*p*-Value
**Age**	29.2 ± 10.3	31.2 ± 15.4	0.572 ^a^
**Gender (%)** **Male** **Female**	16 (64) 9 (36)	27 (61.4) 17 (38.6)	0.828 ^b^
**Duration of illness (days)**	3.9 ± 1	3.9 ± 1.4	0.917 ^a^
**Bleeding Manifestation (%)**	16 (64)	2 (4.5)	**<0.001 ^b^**
**Platelet transfusion done (%)**	24 (96)	11 (25)	**<0.001 ^b^**
**Clinical Features: (%)**			
**Abdominal pain**	2 (8)	10 (22.7)	
**Fluid accumulation**	3 (12)	2 (4.5)	
**Fluid accumulation + Rapid decrease in platelet**	3 (12)	2 (4.5)	0.131 ^b^
**Persistant vomiting**	2 (8)	10 (22.7)	
**Rapid decrease in platelet with rise in hematocrit**	15 (60)	20 (45.5)	
**Type of infection (%)** **Primary (>1.59)** **Secondary (<1.59)**	3 (12) 22 (88)	24 (54.5) 20 (45.5)	**0.001 ^b^**
**Laboratory Findings:**			
**WBC (cells/mm^3^)**	4540 ± 2864.2	4537.8 ± 2683.6	0.997 ^a^
**Packed cell volume (%)**	43.3 ± 5	40.5 ± 5.9	**0.026 ^a^**
**Platelet count (cells/mm^3^)**	40,768 ± 39,267.6	109,504.5 ± 60,163.8	**<0.001 ^a^**
**Haemoglobulin (g/dL)**	14.3 ± 1.9	13.3 ± 1.8	**0.044 ^a^**
**AST (U/L)**	217.1 ± 243.4	104.9 ± 114.9	**0.011 ^a^**
**ALT (U/L)**	124.2 ± 152.8	72.2 ± 95.04	0.085 ^a^
**IL-6 (pg/mL)**	297 ± 116	189.1 ± 125.04	**0.001 ^a^**
**IL-8 (pg/mL)**	351.7 ± 69.1	256.2 ± 85	**<0.001 ^a^**
**Serological test: (%)** **NS1** **IgM** **NS1 + IgG** **NS1 + IgM** **IgM + IgG** **NS1 + IgM + IgG**	0 (0) 0 (0) 14 (56) 0 4 (16) 7 (28)	12 (27.3) 3 (6.8) 17 (38.6) 3 (6.8) 3 (6.8) 6 (13.6)	**0.013 ^b^**
**Dengue serotypes: (%)** **Serotype 1** **Serotype 2** **Serotype 3** **Serotype 4** **Serotypes 2 + 3**	0 (0) 3 (12) 2 (8) 1 (4) 1 (4)	10 (22.7) 7 (15.9) 3 (6.8) 5 (11.4) 0 (0)	**0.049 ^b^**
**APTT (seconds)**	42.4 ± 12.4	35.6 ± 4.6	**0.002 ^a^**
**PT** **(seconds)**	15.1 ± 1.1	16.1 ± 3.1	0.123 ^a^
**INR**	1.1 ± 0.1	1.2 ± 0.2	0.118 ^a^
**Total protein** (g/dL)	6.1 ± 1.02	6.4 ± 0.6	0.104 ^a^
**Albumin** (g/dL)	3.4 ± 0.5	3.6 ± 0.4	**0.023 ^a^**
**Globulin** (g/dL)	2.5 ± 0.6	3.3 ± 4	0.311 ^a^
**A/G ratio**	1.4 ± 0.3	1.4 ± 0.3	0.817 ^a^
**Blood group (%)** **A** **B** **AB** **O**	6 (24) 12 (48) 0 7 (28)	17 (38.6) 14 (31.8) 1 (2.3) 12 (27.3)	0.440 ^b^
**Rh D (%)** **Positive** **Negative**	23 (92) 2 (8)	42 (95.5) 2 (4.5)	0.555 ^b^
**Radiological Findings: (%)**			
**Ascities**	9 (36)	8 (18.1)	0.099 ^b^
**Gallbladder wall thickening**	12 (48)	6 (13.6)	**0.002 ^b^**
**Splenomegaly**	3 (12)	9 (20.5)	0.373 ^b^
**Pleural effusion**	10 (28)	1 (9.1)	**<0.001 ^b^**
**Hepatomegaly**	4 (16)	2 (4.5)	0.105 ^b^
**Hospital stay (days)**	6.2 ± 1.1	5.4 ± 1.4	**0.016 ^a^**

Values in bold indicates statistically significant, *p* < 0.05. ^a^ Levene’s independant *t*-test and ^b^ chi-square test conducted on different variables between Severe dengue and Non-Severe dengue groups.

Follow-up samples (Days 1, 4, and 8) found differences between the Severe and Non-Severe dengue groups. PCV and Hb levels were greater in the Severe group on admission (Day 1), and these differences maintained until follow-up. There is an increase in the concentration of IL-6 and IL-8 during Day 1 and Day 4 in the Severe group when compared to the Non-Severe group, but IL-6 and IL-8 were found to be insignificant in difference on Day 8. Analysis of platelet counts showed that the decreased levels on Day 1 and Day 4 were statistically significant in the Severe group (Table 2).

The dengue serotypes 2, 3, and 4 were found in the both Severe dengue cases and Non-Severe dengue cases, except for co-infection with serotypes 2 + 3, seen only in Severe dengue cases. Dengue serotype 1 was identified only in the Non-Severe dengue cases (Table 1). However, serotypes 2 and 3 were more frequent among platelet-transfused patients (Table S2). AST and ALT were significantly elevated across different dengue serotypes (*p* < 0.001), with higher mean values noted in DEN V2 and DEN V3, and also markedly increases levels in mixed DEN V2 + 3 infections. The levels of IL-8 also demonstrated a statistically significant difference (*p* = 0.04), with DEN V3 cases having the highest levels. Coagulation indicators such as PT and INR were markedly raised (*p* = 0.01), especially in DEN V4. There were high differences in platelet counts between serotypes (*p* = 0.016). DEN V2 had lower counts, while the mixed serotypes had much lower platelet counts. However, the length of hospital stay did not differ much between the groups (Table 3).

**Figure 2:**
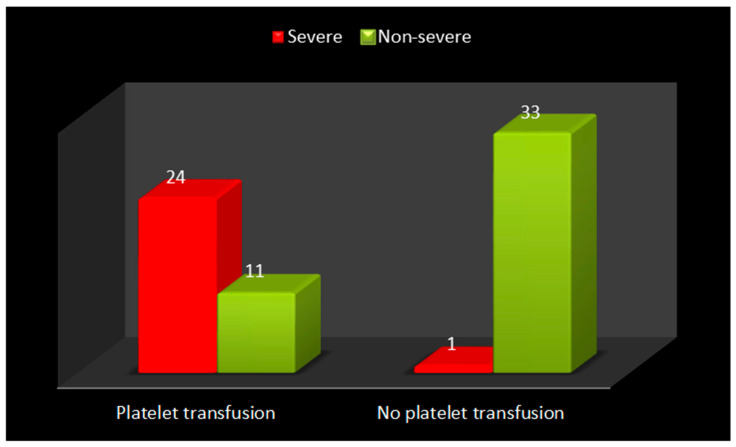
Platelet transfusion among Severe and Non-Severe group.

Among dengue warning sign patients who received platelet transfusions, a higher proportion were classified as severe cases (24 cases) compared to non-severe cases (11 cases) (Figure 2). Overall, dengue with warning signs has increased the length of hospital stays in platelet transfusion cases when compared to cases with no platelet transfusions (Table S3). During the progression of dengue with warning sign cases, there was a significant difference in the levels of Day 1 IL8 and Day 4 IL6 (Table 4).

**Figure 3:**
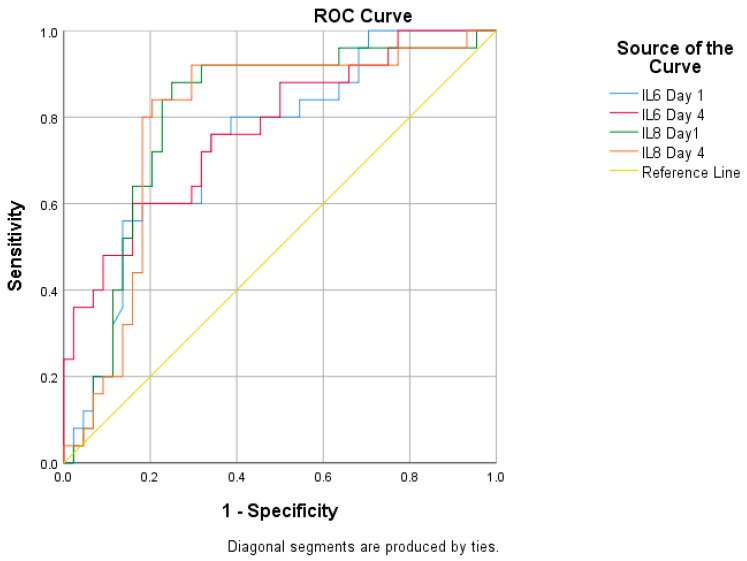
ROC analysis of IL-6 and IL-8 from the Severe dengue group (*n* = 25).

Between the Severe (*n* = 25) and Non-Severe (*n* = 44) dengue cases, IL-6 on Day 1 and Day 4 (AUC = 0.740; 0.772) and IL-8 on Day 1 and Day 4 (AUC = 0.801; 0.788) were found to be significant (Table 5), and a diagrammatic chart representation of the ROC curve analysis is shown in Figure 3, following the prediction for the cut-off value of IL-6 (Day 1 and Day 4 = 194.5 pg/mL; 316.7 pg/mL) and IL-8 (Day 1 and Day 4 = 281.1 pg/mL; 494.1 pg/mL) by the Youden Index (Table S4).

Using the cut-off value, IL-6 and IL-8 were further analyzed through cross-tabulation to identify the odds ratio and *p*-value. To identify the risk of severe dengue, IL-8 and IL-6 were both found significant on Day 1 and Day 4, during the progression of dengue severity (Table 6).

## 4. Discussion

The present study primarily focused on the association between dengue serotypes and laboratory markers in follow-up samples from patients with dengue warning signs. Following the evaluation of patients with dengue who exhibited warning signs, the study aimed to determine whether dengue serotypes and inflammatory markers, specifically interleukin-6 (IL-6) and interleukin-8 (IL-8), could predict the need for platelet transfusion and the progression to severe dengue.

The current study, like Sivasubramanian et al. (2025) demonstrated, shows a similar pattern of laboratory abnormalities among severe dengue patients [21]. The need for platelet transfusion was related to significantly decreased platelet counts, increased packed cell volume, raised AST levels, and a longer aPTT.

A high proportion of secondary dengue infections (88%) were observed from severe dengue patients when compared with the 40% reported in a previous study by Espíndola et al. (2024) [15]. Elevated AST (>40 U/L) and thrombocytopenia (<100,000 cells/mm^3^) were common, consistent with previous findings comparing outpatient and inpatient dengue cohorts. 

In 2022, the Bhattarai et al. study showed that 13.2% were NS1 + IgM-positive with thrombocytopenia, leukopenia, and elevations in AST. Another study found similar results, indicating that 25.1% of patients were NS1 + IgM-positive [22,23]. Our study indicated that severe dengue cases vary by serological profile, with a high frequency of NS1 + IgG-positive patients (56%) and NS1 + IgM + IgG-positive patients (28%). However, NS1 + IgM-positive individuals were not observed in severe dengue cases.

In our study, gallbladder wall thickening (48%) and pleural effusions (28%) were more frequent among Severe dengue patients, and statistically significant when compared with the Non-Severe dengue group. Moras et al. (2022) reported a high prevalence of gallbladder wall thickening (89%) in severe dengue patients, consistent with findings from Xin Tian et al. (2020), who also reported similar findings regarding plasma leakage [24,25]. Shabbir et al. (2018) identified pleural effusion (12.6%) as a marker of severe disease, and Kaagaard et al. (2023) also found it to be consistent with pleural effusion findings in the severity of dengue [26,27].

Prolonged aPTT was significantly associated with severe dengue in our cohort (mean = 43.3 s), consistent with Chi et al. (2023), who reported aPTT > 50 s (36.2%) and liver enzyme elevation > 200 U/L (25.5%) as predictors of high bleeding risk and severe disease [28].

PCV also emerged as a strong predictor of severity. Nandwani et al. (2021) reported that PCV ≥ 45% in combination with platelet count < 20,000 cells/mm^3^ predicted plasma leakage and dehydration (OR = 2.32) [29]. Hemoconcentration due to plasma leakage is a hallmark of severe dengue, underscoring the diagnostic value of PCV. In our follow-up analysis, PCV and hemoglobin were significantly elevated on Day 1, but this significance diminished by Day 4 and Day 8. Conversely, platelet counts remained significantly reduced throughout disease progression, but, on Day 8, platelet counts reverted back to the normal range.

Prolonged elevation of IL-8 has been associated with dengue immunopathogenesis, which plays a role in disease severity by contributing to plasma leakage and severe manifestations, and IL-6 directly activates vascular endothelial cells through trans-signalling to produce pro-inflammatory cytokines [30,31]. These findings suggest that IL-8 exhibited greater discriminative ability than IL-6 in predicting severe dengue, particularly during the early phase. These results indicate that both early elevation and persistence of IL-6 and IL-8 mirror an exaggerated inflammatory response that contributes to vascular leakage and disease progression.

Comparable observations were made by Prajapati et al. (2024) [14], who reported that IL-6 (OD = 0.909; AUC = 0.744) predicted 74% of severe dengue cases, and other studies found similar findings for the prediction of severe dengue [32]. In our study, IL-6 demonstrated predictive accuracy (68%) using a cut-off of 194.5 pg/mL, but IL-8 had even better predictive accuracy (76.8%) at the cut-off value of 281.1 pg/mL; also, IL-8 is an earlier predictor of severe dengue than IL-6 by the analysis of length of hospital stay. The meta-analysis study done by Moallemi et al. (2023) mentioned that IL-8 is one of the predictors for severe dengue, and Jiravejchakul et al. (2025) also found elevated IL-8 levels at the acute phase in severe dengue when compared to non-severe dengue [30,33].

Our serotype distribution in 32 cases observed DEN V-1 (31.3%), DEN V-2 (31.3%), DEN V-3 (15.6%), DEN V-4 (18.8%), and mixed DEN V 2 + 3 (3.1%). This pattern reflects the hyperendemic circulation of multiple serotypes in India. Previous studies from northern India indicated a simultaneous circulation of DENV-1, DENV-2, and DENV-3, with shifts in the dominant serotype found across successive outbreaks. In contrast, studies from South India have shown the circulation of all four dengue serotypes, with DENV-4 and DENV-2 reported with significantly greater frequency [8].

The key laboratory parameters, including platelet count, AST, ALT, IL-8, PT, and INR, among dengue patients with warning signs, for DEN V-2 and co-infected with DEN V-2 + 3 showed highly significant differences compared to other serotypes. These findings underscore the clinical heterogeneity of dengue infection and suggest that circulating serotypes may differentially influence hematological, hepatic, inflammatory, and coagulation profiles.

A significant difference in platelet count across serotypes was observed in our study. Thrombocytopenia remains a hallmark of dengue pathogenesis, and may result from bone marrow suppression, immune-mediated platelet destruction, and peripheral consumption [10]. Previous studies from India and Sri Lanka have shown that DEN V-2 and DEN V-3 are more commonly associated with severe thrombocytopenia and increased clinical severity [9,10], and, similarly, our cohort demonstrated that infections with DEN V-2, DEN V-3, DEN V-4, and mixed DEN V-2 + 3 were linked to a significantly greater reduction in platelet counts compared to DEN V-1, suggesting a potential role of antibody-dependent enhancement.

Hepatic involvement, reflected by elevated AST and ALT levels (*p* < 0.001), showed significant variation between DEN V-2 and DEN V-2 + 3 serotypes in our study. Liver dysfunction in dengue may arise from direct viral hepatocyte injury, immune-mediated damage, and hypoxic insult during plasma leakage. A study from North India demonstrated that biochemical markers (AST and ALT) were significantly associated with the disease severity in DEN V-2 individuals [6,7,10]. The observed serotype-specific differences in AST and ALT in our cohort suggest that viral strain variability may influence the extent of hepatic injury, reinforcing the importance of routine liver function monitoring in patients with warning signs.

Notably, IL-8 levels varied significantly across serotypes. Cytokine dysregulation is central to dengue pathogenesis, particularly in mediating endothelial dysfunction and vascular permeability. During the South Indian outbreak in which DEN V-4 emerged as the dominant serotype, altered IL6 cytokine expression profiles were documented, especially of DEN V-2 when compared to other serotypes, indicating that serotype shifts may modify inflammatory responses [8]. In our study, IL-6 levels did not have a statistically significant relationship with dengue serotypes.

Significant variations were identified among patients who received platelet transfusions among dengue serotypes, especially in DENV-2 and DENV-3. The coagulation parameters (PT and INR) (*p* = 0.01) also demonstrated significant differences in serotypes. Prolonged PT and elevated INR reflect hepatic dysfunction and consumptive coagulopathy, both of which predispose patients to bleeding complications. Our findings suggest that certain serotypes may exert a greater impact on coagulation pathways, highlighting the need for close monitoring of coagulation profiles in serotype-prevalent outbreaks.

## 5. Conclusions

The study indicates that IL-8 serves as an earlier prediction biomarker than IL-6 for detecting dengue patients susceptible to advancement to severe dengue. Early monitoring and monitoring during the progression of these cytokines can help clarify the difference between severe and non-severe cases and contribute to better decisions about platelet transfusion support in severe dengue. Significant associations are found between dengue serotypes and crucial laboratory findings. DENV-2 and mixed DENV-2 + 3 infections were especially associated with severe thrombocytopenia and may be influenced by small subgroup sizes and requires validation in larger cohorts, high AST and ALT levels, raised IL-8 levels, and a prolonged PT/INR. Overall, combining cytokine testing with serotype testing and lab monitoring should help clinicians better identify the risk of dengue patients with warning signs during the follow-up and manage individuals more effectively in dengue endemic regions.

## Figures and Tables

**Table 2:** Correlation of follow-up samples’ (Day 0, 4, 8) laboratory markers between the Severe dengue and Non-Severe dengue groups and dengue serotypes.

Parameter		Severe Dengue (*n* = 25)	Non-Severe Dengue (*n* = 44)	DEN V1 (*n* = 10)	DEN V2 (*n* = 10)	DEN V3 (*n* = 5)	DEN V4 (*n* = 6)	DEN V2 + 3 (*n* = 1)
WBC (cells/mm^3^) Normal range (4000–11,000 cells/mm^3^)	Day 1 (*n* = 69)	4540 ± 2864.2	4538 ± 2683.6	4439 ± 2198.5	4905 ± 4310.3	4144 ± 1350.9	2636.6 ± 386.8	6680
	*p*-value	0.997 ^a^		0.549 ^b^				
	Day 4 (*n* = 69)	6920 ± 2663.2	5723.5 ± 3022.5	4575 ± 1951.5	6817 ± 4203.6	6256 ± 1728.7	5705 ± 2756.2	10,380
	*p*-value	0.104 ^a^		0.332 ^b^				
	Day 8 (*n* = 20)	6850 ± 1599	5715.5 ± 1602.6	6012.5 ± 864.4	5550 ± 2899.1	6640 ± 1827.4	5025 ± 176.8	6790
	*p*-value	0.148 ^a^		0.921 ^b^				
PCV (%) Normal range (male, 40–50%; female, 36–46%)	Day 1 (*n* = 69)	43.3 ± 5.02	40.5 ± 4.8	40 ± 4	41.1 ± 4.1	42.6 ± 4.7	42.2 ± 2.6	44
	*p*-value	**0.026 ^a^**		0.912 ^b^				
	Day 4 (*n* = 69)	41 ± 6.2	41 ± 5.1	42.5 ± 4.5	39.3 ± 3.2	44 ± 5.6	42.8 ± 2.6	35
	*p*-value	0.997 ^a^		0.374 ^b^				
	Day 8 (*n* = 20)	40.3 ± 5.4	40.7 ± 6	42.5 ± 5.7	38 ± 2.8	43 ± 1	47 ± 2.8	33
	*p*-value	0.883 ^a^		0.240 ^b^				
HB (g/dL) Normal range (male, 13–17 g/dL; female, 12–15 g/dL)	Day 1 (*n* = 69)	14.3 ± 1.9	13.3 ± 1.8	(13.1 ± 1.6)	(13.5 ± 1.7)	(14 ± 2.1)	(13.9 ± 1)	(14.8)
	*p*-value	**0.044 ^a^**		0.915 ^b^				
	Day 4 (*n* = 69)	13.4 ± 2	13.3 ± 1.8	(13.7 ± 1.9)	(12.9 ± 1.3)	(14.2 ± 2.1)	(13.9 ± 1)	(11.3)
	*p*-value	0.916 ^a^		0.552 ^b^				
	Day 8 (*n* = 20)	13.1 ± 1.8	13.5 ± 2.2	(13.9 ± 2.3)	(12.5 ± 1.1)	(14.3 ± 0.4)	(15.6 ± 1.1)	(11.5)
	*p*-value	0.749 ^a^		0.351 ^b^				
Platelet count (cells/mm^3^) Normal range (150,000–410,000 cells/mm^3^)	Day 1 (*n* = 69)	40,768 ± 39,267.6	109,504.5 ± 60,163.8	(146,120 ± 60,670.3)	(74,100 ± 63,356)	(87,320 ± 47,891.9)	(93,366.7 ± 40,224.1)	(9400)
	*p*-value	**<0.001 ^a^**		**0** **.016 ^b^**				
	Day 4 (*n* = 69)	50,864 ± 34,541.8	89,200 ± 56,686.9	(81,960 ± 57,522.9)	(91,300 ± 55,079.8)	(87,440 ± 51,823)	(59,133.3 ± 37,290.1)	(37,200)
	*p*-value	**0.003 ^a^**		0.769 ^b^				
	Day 8 (*n* = 20)	119,600 ± 41,080.7	92,307.7 ± 43,025.9	(82,800 ± 20,832)	(140,200 ± 49,214.6)	(148,266.7 ± 60,384.2)	(76,700 ± 3252.7)	(157,400)
	*p*-value	0.186 ^a^		0.060 ^b^				
IL-8 (pg/mL)	Day 1 (*n* = 69)	351.7 ± 69.1	256.2 ± 84.7	(237 ± 79.3)	(285.2 ± 86.4)	(308.2 ± 75.1)	(227.2 ± 96.8)	(454.8)
	*p*-value	**<0.001 ^a^**		**0** **.045 ^b^**				
	Day 4 (*n* = 69)	578.5 ± 98	458.3 ± 124.8	(422.6 ± 54.5)	(510.9 ± 122.4)	(600.4 ± 128.3)	(423.9 ± 135.7)	(542.7)
	*p*-value	**<0.001 ^a^**		0.078 ^b^				
	Day 8 (*n* = 20)	565 ± 89.2	453.9 ± 227.3	(312.1 ± 196.9)	(401.4 ± 214.9)	(696.5 ± 82)	(516.8 ± 322.2)	(522.7)
	*p*-value	0.131 ^a^		0.185 ^b^				
IL-6 (pg/mL)	Day 1 (*n* = 69)	297 ± 116	189.1 ± 125	(188.3 ± 116.6)	(220.4 ± 129.7)	(233.8 ± 115.3)	(157.6 ± 89.2)	(377.3)
	*p*-value	**0.001 ^a^**		0.455 ^b^				
	Day 4 (*n* = 69)	360.5 ± 99.3	242.2 ± 118.8	(259.4 ± 128.4)	(262.9 ± 148.1)	(320 ± 99.7)	(299.3 ± 89.6)	(510.4)
	*p*-value	**<0.001 ^a^**		0.500 ^b^				
	Day 8 (*n* = 20)	176 ± 80.7	209.6 ± 73	(175.8 ± 113.1)	(192.6 ± 2.4)	(192.9 ± 36.2)	(242.9 ± 33.4)	(195.5)
	*p*-value	0.357 ^a^		0.969 ^b^				

Values in bold indicates statistically significant, *p* < 0.05. ^a^ Levene’s independent *t*-test conducted on different variables between the Severe dengue and Non-Severe dengue groups; ^b^ one-way ANOVA test between the follow-up variables and dengue serotypes.

**Table 3:** Influence of dengue serotypes with laboratory markers.

Parameters	DEN V1 (*n* = 10)	DEN V2 (*n* = 10)	DEN V3 (*n* = 5)	DEN V4 (*n* = 6)	DEN V2 + 3 (*n* = 1)	*p*-Value
**AST (U/L)**	(96 ± 91.7)	(180.3 ± 204.1)	(173.4 ± 211.1)	(134.8 ± 113.4)	(1088)	**<0.001 ^#^**
**ALT (U/L)**	(55.4 ± 74.6)	(122.3 ± 157.5)	(89 ± 108.6)	(93.8 ± 102.9)	(618)	**<0.001 ^#^**
**IL-8 (pg/mL)**	(216.3 ± 49.7)	(284.4 ± 85.6)	(357.5 ± 94.5)	(219.6 ± 75.2)	(334.6)	**0.04 ^#^**
**PT (seconds)**	(15.4 ± 1.6)	(15.9 ± 2.5)	(15 ± 0.5)	(19.4 ± 6.3)	(14.1)	**0.01 ^#^**
**INR**	(1.1 ± 0.1)	(1.1 ± 0.2)	(1.1 ± 0.04)	(1.4 ± 0.5)	(1.01)	**0.01 ^#^**
**Platelet count (cells/mm^3^)**	(146,120 ± 60,670.3)	(74,100 ± 63,356)	(87,320 ± 47,891.9)	(93,366.7 ± 40,224.1)	(9400)	**0.016 ^#^**
**Hospital stay**	(5.6 ± 1.5)	(6.1 ± 1.3)	(6.2 ± 1.8)	(5.8 ± 1.3)	(8)	0.437 ^#^

^#^ One-way ANOVA test, Values in bold indicates for *p* < 0.05 as the level of significance (laboratory markers against dengue serotypes).

**Table 4:** Effect of length of hospitalization on IL-6 and IL-8 levels.

Parameter	Length of Hospitalization (*n* = 69)	Significance (*p*-Value)
**IL-6 Day 1**	5.7 ± 1.3	0.260 ^c^
**Day 4**		**<0.001 ^c^**
**Day 8**		0.280 ^c^
**IL-8 Day 1**		**0.021 ^c^**
**Day 4**		0.321 ^c^
**Day 8**		0.197 ^c^

Values in bold indicates statistically significant, *p* < 0.05. ^c^ Pearson correlation test.

**Table 5:** ROC analysis of IL-6 and IL-8 from the Severe dengue group (*n* = 25).

Parameters	AUC	*p*-Value	95% of C.I	
IL-6 Day 1	0.740	0.001 ^e^	0.621	0.860
IL-6 Day 4	0.772	<0.001 ^e^	0.656	0.887
IL-8 Day 1	0.801		0.688	0.914
IL-8 Day 4	0.788		0.671	0.906

^e^ Receiver operating characteristic (ROC) curve.

**Table 6:** Using the optimum cut-off value of IL-6 and IL-8 for the risk of severe dengue.

Parameter	Cut-off Value	Odds Ratio & *p*-Value	95% of C.I
IL-6 Day 1	>194.5 = 37 <194.5 = 32	6.353 **0.001 ^d^**	2.006–20.117
IL-6 Day 4	>316.7 = 34 <316.7 = 35	6.122 **0.001 ^d^**	2.019–18.568
IL-8 Day 1	>281.1 = 37 <281.1 = 32	24.643 **<0.001 ^d^**	5.086–119.405
IL-8 Day 4	>494.1 = 36 <494.1 = 33	27.423 **<0.001 ^d^**	5.629–133.592

Values in bold indicates statistically significant *p* < 0.01. ^d^ Fisher’s exact test.

## Data Availability

The original contributions presented in this study are included in the article/Supplementary Materials. Further inquiries can be directed to the corresponding author.

## Supplementary Materials

The following supporting information can be downloaded at: https://www.mdpi.com/article/10.3390/pathogens15040434/s1. Table S1: Classification of Severe dengue (*n* = 25) cases based on the WHO 2009 guidelines; Table S2: Correlation of Dengue serotypes against Platelet transfusion & bleeding manifestation; Table S3: Correlation of Platelet transfused cases and No platelet transfusion cases from the length of Hospital stay; Table S4: Prediction Cut-off value by Youden Index for IL6 and IL8.
