# Supplementary material for: Assessment of IL-6 and IL-8 Levels and Other Bio Markers in Predicting Dengue Severity Across Serotypes
Source: Pathogens. 2026 Apr 17;15(4):434. doi: 10.3390/pathogens15040434 (PMC13119028; doi:10.3390/pathogens15040434)
Supplement: Supplementary file 1 [file pathogens-15-00434-s001.zip › pathogens-4225117-supplementary.pdf]

Table S1. Classification of Severe dengue (n=25) cases based on the WHO 2009 guidelines

Criteria for Severe dengue	No. of. Severe dengue cases
**Elevation of Haematocrit + Bleeding**	16 (64%)
**Plasma leakage - Pleural effusion + Ascites & Low albumin level**	9 (36%)

Table S2. Correlation of Dengue serotypes against Platelet transfusion & bleeding manifestation

Dengue serotypes	Platelet transfusion		Bleeding manifestation	
	Yes	No	Yes	No
**Dengue 1 (10)**	1	9	1	9
**Dengue 2 (10)**	4	6	2	8
**Dengue 3 (5)**	4	1	2	3
**Dengue 4 (6)**	1	5	2	4
**Dengue 2+3 (1)**	1	0	0	1
**p-value**	**.008[b]**		.730[b]	

[b]Chi- square test, p <.05 at the level of significant (Dengue serotypes- Platelet transfusion Vs No platelet transfusion; Bleeding Vs No bleeding)

Table S3. Correlation of Platelet transfused cases and No platelet transfusion cases from the length of Hospital stay

Dengue with warning sign (n=69)	Platelet transfusion (n =35)	No platelet transfusion (n=34)	P-value
**Length of Hospital stay (days)**	6.09 ± 1.4	5.35±1.6	**.023[a]**

Statistically significant, p<0.05 [a] between the Platelet transfused cases and length of Hospital stay

Table S4. Prediction Cut-off value by Youden Index for IL6 and IL8

Marker	Day	Optimal Cutoff (≥ pg/mL)	Max Youden Index
**IL-6**	Day 1	194.5	0.414
	Day 4	316.7	0.419
**IL-8**	Day 1	281.1	0.602
	Day 4	494.1	0.625
